# OSCEs are outdated: clinical skills assessment should be centred around workplace-based assessments (WPBAS) to put the ‘art’ back into medicine

**DOI:** 10.15694/mep.2017.000189

**Published:** 2017-10-23

**Authors:** Hamed Khan

**Affiliations:** 1 St Georges

**Keywords:** OSCE, Assessment, Clinical Skills, Workplace based assessments, Medical Education

## Abstract

This article was migrated. The article was marked as recommended.

OSCEs have gradually replaced ‘long cases’ as the mainstay of undergraduate clinical skills assessment because of their objectivity, consistency and reliability. But the aspects of OSCEs which make them so reliable increasingly encourage students to prepare strategically, who often adopt a robotic ‘tickbox’ approach, rather than use OSCEs as a tool to learn clinical skills for safe competent real-life practice.

Thus, whilst OSCEs facilitate technical competence, they do not prepare students for the unique nuances that make medicine an ‘art’ as well as a science.

In pursuit of consistency and reliability, we are sacrificing validity and not preparing future doctors for the innate nuances and variability that make medicine so unique- and which often come as a shock to newly qualified doctors who orientate their undergraduate learning around OSCEs rather than real life.

The doctors of the future will need to be adaptable and be able to vary their practice depending on the clinical and biopsychosocial context much more so than before. To drive their learning accordingly, we need a paradigm shift in medical education and assessment. WPBAs should now take centre‐stage in undergraduate clinical assessment, with OSCEs significantly scaled back.

## OSCEs are outdated: clinical skills assessment should be centred around workplace-based assessments (WPBAs) to put the ‘art’ back into medicine

I love OSCEs. I have been fascinated by them since I started medical school. As a student, I voluntarily spent several hours of my spare time setting up mock OSCEs for fellow students, when I should have been studying myself. After I qualified, I became an OSCE examiner for 3 different London medical schools. Later in my career, I wrote an OSCE textbook. Now, at the pinnacle of my career as a Lecturer in Clinical Skills, I am in charge of running an OSCE for two entire cohorts of students at a prominent London medical school, doing what was always my ‘dream job’.

So I have been immersed in OSCEs all my life- and have thoroughly enjoyed it. Setting up an OSCE, watching its mechanics roll into action asintelligent but terrified students demonstrate their skills and knowledge, and then seeing that translate into a structured report on their performance, still gives me unparalleled joy.

However over the last few years, my love affair with OSCEs has soured. I have become increasingly sceptical about how well OSCEs prepare students for the trials and tribulations of real life practice. I reluctantly concluded that OSCEs were no longer fit for purpose when I met one of former students who had just graduated. He was a brilliant hard working insightful student, so I was surprised when he told me that he was terrified of starting work as a doctor. When I asked him why, he gave me an interesting answer- he said that all of his life, he had been learning clinical skills to pass OSCEs- and now, he needed to learn clinical skills for real life.

Firstly, one cannot understate the decisively positive impact that OSCEs have had on clinical learning. Decades ago clinical skills assessment was based around ‘long cases’, with relatively open-ended instructions, examiners who could ask questions and conduct vivas as they wished to. The inevitable examiner bias led to significant unreliability.

I imagine that this unreliability drove the evolution from ‘long cases’ to the modern day OSCE, with specific tasks and instructions for candidates, and clear objective marking criteria for examiners. Both candidates and examiners are briefed carefully, and academics work relentlessly to ensure consistency and reliability.

But in my opinion, the pendulum has swung too far. As academics, our well intentioned focus on process has led us to neglect crucial outcomes, and we are losing the forest for the trees.

Our focus on reliability, and making OSCEs as objective and structured as possible, has led to a paradigm where students know what to expect. There is little variation amongst OSCE scenarios both within and across medical schools, and OSCE textbooks and resources contain very similar content. Unsurprisingly, students largely orientate their learning strategically to pass OSCEs, often adopting a robotic ‘tickbox’ approach. The idea of using OSCEs as a tool for meaningful learning to become safe competent practitioners who are able to assess and treat real patients rarely motivates students- as demonstrated by my example above.

Hence our students gain technical competence from OSCEs, but struggle to translate this into skills which are practically useful in the dynamic, ever changing environment of real frontline medicine. The A&E department with several acutely sick patients presenting simultaneously, or the inner city GP practice where doctors deliver holistic care for patients whilst disentangling medical issues from psychosocial factors, is far removed from the comfortable confines of an OSCE station, where simulated patients always stick to the script, and candidates never really need to expect the unexpected. The gap between the ‘Shows How’ of Millers Pyramid, and ‘Does’ in clinical practice is getting increasingly steeper. In real life, a plethora of variables intertwine with pathology to create the unique dynamic of individual scenarios and consultations- such as psychosocial factors, patient perspectives, time and resource pressures, in addition to natural variation amongst individual humans. Whilst OSCEs help students attain basic competence in clinical skills, they fail to prepare them for the complex nuances that make medicine unique amongst disciplines, as both an ‘art’ as well as a science. If anything, these nuances will only become more complex for the doctors of the future, as they face unique challenges- aging populations with complex medical problems intertwined with intractable social issues, resource constraints, and an unprecedented increase in access to information, leading to patients who are better informed, and treatment guidelines and pathways which change on a daily basis.

Thus whilst technical competence will remain crucial, the doctors of the future will also need to be able to adapt and improvise their skills depending on the situation, much more so than ever before. They will need to be able to innovate and think outside the box, both on the clinical front line, as well as outside it as leaders and managers of their health services.

In my opinion, whilst we have made vital progress with assessment reliability from OSCEs, we are sacrificing validity to an unacceptable extent, and leaving our future doctors unprepared for the innate nuances and variability that make medicine unique.

So what is the answer? A key overarching principle of high quality assessment is having a variety of both assessments and assessors. But more specifically, I feel that a paradigm shift is needed where we put workplace-based assessment (WPBA) at the centre of clinical skills assessment, and significantly reduce the role of OSCEs. WPBAs such as CEXs and CBDs are used extensively for postgraduate assessment. They allow us to assess both technical competence, as well as the ability of students to adapt skills, and improvise and innovate, and vary their practice depending on the individual dynamic and context of the scenario. Using WBPAs also enforces a vital ‘hidden curriculum’ message that the ultimate aim of learning clinical skills is for real life practice with real patients, rather than confining it to artificial simulated settings, and thus change the way students think- both individually and collectively. Students will feel compelled to think more about how they would realistically conduct a cardio-respiratory exam in an elderly lady who is short of breath in A&E, and less about the causes of clubbing and janeway lesions- which are rarely seen in practice, but seem to be crucially important for OSCEs. Over the course of time, those with a more collateral role in student education, such as textbook authors, private course organisers, societies etc, will also change their approach to students, and a paradigm shift will slowly occur.

Whilst I am advocating a reduced role for OSCEs, I am absolutely not suggesting that they be discarded altogether. OSCEs play a vital role in assessing basic technical competences, and the reliability that they bring to medical assessment overall is also crucially important.

I also recognise the drawbacks of WPBAs, such as interassessor variability, and the logistical complexities of conducting assessments on a large scale in ‘live’ clinical settings- but we have a precedent. WPBAs were introduced, and are now used extensively in postgraduate training and assessment in the UK. The Royal College of General Practitioners, which sets the assessments used to certify the completion of GP training, uses WPBAs extensively, and GP training is now unimaginable without them. This is particularly noteworthy as most care in the British NHS is delivered through GPs, which is expected to be the end career point for most medical students. Surely we can look at what similar postgraduate training organisations have done, and use, adapt and improve on their work and their existing infrastructures, to build similar frameworks for undergraduate clinical assessment?

At the very least, we need to start dedicating time, thought and resources towards developing frameworks and educators with the aim of making WPBAs much more integral to clinical skills than they are now. The debate around clinical skills assessment has stagnated, and the perception that OSCEs are the panacea of clinical skills assessment is increasingly unquestioned. We need to reopen that discussion, and start pushing its boundaries in order to reignite the evolution which led to the formation of OSCEs in the first place. It is high time that we used WPBAs to compel our students to step outside the comfort zones of surreal OSCE settings, and learn and practice their clinical skills to excel in real life settings, with real patients, in real clinical scenarios.

## Take Home Messages

• OSCEs are outdated, and encourage strategic OSCE orientated learning, rather than ‘deep’ meaningful learning to become safe competent clinicians.
• In our well-intentioned pursuit for reliability, we have made huge sacrifices in validity. OSCEs no longer prepare students for the innate nuances that make medicine so unique as both an ‘art’ as well a ‘science’.
• Factors such as aging populations, financial constraints, and ever improving access to information (for both patients and doctors) mean that future doctors will need ‘real life’ skills such as the ability to adapt, improvise and innovate more so than ever before- both as service providers as well as managers and leaders of their health services.
• Workplace based assessment allow us to assess students ability to do this as they are centred around real life patients in real life settings, rather than in the comfort zone of simulated OSCE settings.
• It is time for academics in undergraduate medicine to take a leap of faith, and work towards making WPBAs the mainstay of clinical skills assessment, and scale back the role of OSCEs.

## Notes On Contributors

Dr Hamed Khan is a Lecturer in Clinical Skills and Responsible Examiner for the Clinical Sciences OSCE at St Georges, University of London. He has been an OSCE examiner at three London medical schools, and has written a textbook on OSCEs for medical finals ( http://eu.wiley.com/WileyCDA/WileyTitle/productCd-0470659416.html). In addition to this, he is a practising GP in South London, and is a National Council member of the British Medical Association. He has also written on NHS related issues for the Guardian and the Channel 4 websites.

## Declarations

I am a Lecturer in Clinical Skills at St Georges (University of London) and the Responsible Examiner for the Clinical Sciences OSCE. I am also a GP in London. I have co-authored a textbook on OSCEs for Medical Finals. I presented a ‘Point of View’ oral presentation at the AMEE 2017 Conference.

