# Supplementary material for: OSCEs are outdated: clinical skills assessment should be centred around workplace-based assessments (WPBAS) to put the ‘art’ back into medicine
Source: MedEdPublish (2016). 2017 Oct 23;6:189. [Version 1] doi: 10.15694/mep.2017.000189 (PMC10885310; doi:10.15694/mep.2017.000189)
Supplement: Peer review history

# Reviewer report: Reviewer response for version 1

**DOI:** 10.21956/mep.19377.r28379

**Published:** 2018-09-29

**Authors:** Anthony David.M

**Affiliations:** 1 Soho

This review has been migrated. The reviewer awarded 4 stars out of 5 I enjoyed reading the opinion piece and the interesting reviews too. Indeed the author is passionate and presents his case very well. It is a fact that the Global assessment is sadly lacking in OSCEs. The OSCEs have come in as a reaction to the subjective and unreliable methods of clinical assessment which existed in the form of long and short cases. But it has it's own pitfalls. Workplace based assessment methods seems to be the more suitable method. But as some earlier reviewers opined, an eclectic approach using more than one method of assessment may be best.

Reviewer Expertise:

NA

No decision status is available

# Reviewer report: Reviewer response for version 1

**DOI:** 10.21956/mep.19377.r28385

**Published:** 2018-04-07

**Authors:** Nandalal Gunaratne

**Affiliations:** 1 Faculty of Medicine, Wayamba University of Sri Lanka

This review has been migrated. The reviewer awarded 3 stars out of 5 The Title of the article is quite striking but the article itself shows a more balanced opinion from the author. Perhaps we could lend more time to improving and expanding the OSCE.WPBA has its own pitfalls and is not as suitable for assessing undergraduates as it is in assessing the postgraduate. This is because the undergraduate is not a "real doctor"and has a different relationship with both patients and staff.

Reviewer Expertise:

NA

No decision status is available

# Reviewer report: Reviewer response for version 1

**DOI:** 10.21956/mep.19377.r28381

**Published:** 2017-11-05

**Authors:** Alan Jaap

**Affiliations:** 1 University of Edinburgh Medical School

This review has been migrated. The reviewer awarded 2 stars out of 5 Thank you for this thought-provoking commentary which has unsurprisingly stimulated a healthy debate on the difficult area of how to assess clinical performance authentically while making best use of the limited resources available.The author highlights the problems associated with relatively simple OSCE stations and check list marking. However, many would argue that the educational impact of such an approach is actually quite positive for early clinical learners, and, as other reviewers have already stated, the complexities of both station design (e.g. integrated skills) and marking schedules (e.g. domain based) can be increased to make the assessment more authentic as students progress towards graduation.The problems of existing WPBAs have also been outlined by several other reviewers, and I would simply add that it remains difficult to ensure consistent sampling of learning outcomes in the workplace, as by their nature these WPBAs are used opportunistically. A move to more longitudinal assessments in the workplace, for instance through the introduction of a framework of Entrustable Professional Activities, may offer a feasible solution to many of the issues raised going forward.

Reviewer Expertise:

NA

No decision status is available

# Reviewer report: Reviewer response for version 1

**DOI:** 10.21956/mep.19377.r28388

**Published:** 2017-11-03

**Authors:** Gerard Flaherty

**Affiliations:** 1 National University of Ireland Galway

This review has been migrated. The reviewer awarded 3 stars out of 5 The author provides a timely commentary on the future role of workplace-based assessment in undergraduate clinical skills education. His credibility stems from his vast experience in OSCE station design and in OSCE coordination and delivery. While I agree with his sentiments about the strategic learning which OSCEs can inspire, I wonder if he is being unfair on students, whose motivation probably extends well beyond merely preparing for OSCEs. Our challenge in clinical skills assessment is to maximise the value of OSCEs so as to encourage deep learning, and to remove the predictability which the author criticises. In this respect, it behoves us to collaborate in designing more authentic stations, perhaps of longer duration, and in utilising simulation-based assessment to a greater extent. This would enable us to assess with greater sophistication students' human factors skills, including situational awareness and multi-tasking. This is happening to varying extents already in different medical schools, but I believe there is untapped potential in sharing best OSCE design practice. Perhaps the model has become stale and has lots its creativity. OSCEs remain a very useful, efficient and reliable tool for undergraduate clinical skills assessment, but we need to do more to adapt them to changing healthcare needs, by designing more complex, authentic and challenging stations. This opinion paper is thought-provoking and may be a stimulus for more original research in collaborative OSCE station design, and I therefore welcome its submission.

Reviewer Expertise:

NA

No decision status is available

# Reviewer report: Reviewer response for version 1

**DOI:** 10.21956/mep.19377.r28377

**Published:** 2017-11-02

**Authors:** Ralph Pinnock

**Affiliations:** 1 University of Orago, Dunedin, New Zealand

This review has been migrated. The reviewer awarded 3 stars out of 5 I have a lot of sympathy with the views expressed by Dr Khan but wonder whether the answer is not ‘yes’ to both forms of assessment as components of programmatic assessment. OSCEs are not easy to organise in children. Although there publications of using children as simulated patients in OSCEs to assess medical students’ clinical skills, they can be difficult to organise. How many candidates can a well 5 year old child be expected to see? When I became responsible for a paediatric curriculum where I previously worked we changed the in course assessment from an end of rotation OSCE to 5 miniCEXs during the rotation. We used patients on our inpatient service because we wanted to assess our students’ clinical skills with children who had abnormal physical signs. Our students were often reluctant to see children who had been admitted acutely citing the stress that both mother and child would be experiencing. To try and allay students concerns we interviewed mothers and children to find out their attitude to seeing medical students (Pinnock R, Weller J, Shulruf B, Reed P, Mizutani S. Why parents and children agree to become involved in medical student teaching. (J. Paediatr.Child Health 2011;47(4):204 -210). I was surprised how even children as young as 6 or 7 were willing to see and be examined by students so that “doctors can learn to help other children”. It was always necessary to see the parent and child after the miniCEX because almost invariably the parent had become concerned by some of the students comments usually, related to including a very serious disorder in the differential diagnosis. One very valuable insight gained from this is work-placed base assessment into the student’s performance was to ask the parent and child if they would see that “doctor” again. There are certainly advantages to work-based assessments.

Reviewer Expertise:

NA

No decision status is available

# Reviewer report: Reviewer response for version 1

**DOI:** 10.21956/mep.19377.r28380

**Published:** 2017-11-01

**Authors:** Julie Hunt

**Affiliations:** 1 Lincoln Memorial University

This review has been migrated. The reviewer awarded 2 stars out of 5 As a clinical skills educator who creates OSCE materials, I read this opinion piece with interest. The author proposes that OSCEs be eschewed in favor of WPBA which he suggests have higher validity albeit lower reliability. As previous reviewers have stated, I would have liked to have seen references in support of some of his points. Different forms of assessment have varying strengths and weaknesses in terms of feasibility, reliability, validity, richness of feedback, and suitability for different levels of learners. Assessments of multiple different types probably give us the most complete view of a student's competence, as has been proposed by van der Vleuten.

Reviewer Expertise:

NA

No decision status is available

# Reviewer report: Reviewer response for version 1

**DOI:** 10.21956/mep.19377.r28387

**Published:** 2017-10-26

**Authors:** Richard Hays

**Affiliations:** 1 James Cook University

This review has been migrated. The reviewer awarded 2 stars out of 5 While I found this opinion paper entertaining and thought provoking, I wonder the author has been distracted by popularism rather than theory. OSCEs play an important role in demonstrating that, at a particular point in time, candidates demonstrate the ability to perform tasks that are essential components of future practice. It is possible to go beyond this point, with more elaborate simulations and tasks. Workplace based assessment is really one of those ‘holy grail’ issues. Yes, what candidates actually do in the workplace in ultimately more important, and should be part of the assessment package, but there are many pitfalls. The first is that scores have been shown to be inflated, with very similar rank ordering, and so standard setting is more difficult. Most achieve what appear to be high scores, although norm referencing can be helpful. Second, working with colleagues whom we assess raises the likelihood that judgements are based on global, halo impressions of being ‘nice’ and ‘good’, or possibly the opposite. This is where validity and reliability have the potential to collide. The third problem is the answer to the last point. WBA requires multiple judges, multiple assessments, multiple methods on multiple occasions and from different perspectives. This is just really hard to do well. If we are not careful, we will replace OSCEs with narrowly based judgements by colleagues, which would be almost full circle in the development of assessments in medical education. All very thought provoking, but there is unlikely to be any simple solution.

Reviewer Expertise:

NA

No decision status is available

# Reviewer report: Reviewer response for version 1

**DOI:** 10.21956/mep.19377.r28383

**Published:** 2017-10-25

**Authors:** P Ravi Shankar

**Affiliations:** 1 American International Medical University

This review has been migrated. The reviewer awarded 4 stars out of 5 I read with great interest this thought provoking paper. When I was in medical school, long cases and short cases were the mainstay of clinical exams. Long cases may more accurately reflect the variability and the nuances of medical practice but the major problem was variability in grading among examiners, differences among patients and among diseases. The trend toward OSCE came about primarily because of the need for objectivity and reliability. For high stakes exams it became important to reduce bias and variation. The premise was the process of medical consultation could be broken down into various component parts which could be examined separately and objectively. However, many authors now think that this may be an oversimplification. Caribbean medical schools mainly prepare students for a career in the United States and Canada and OSCEs and standardized patients are important methods of assessment. Standardizing variables in the patient-doctor consultation makes assessment more standardized and reliable but as the author mentions the variability and the ‘richness’ of a real-life consultation may be missing. WPBA may be a good alternative and has been widely used in postgraduate education. This may however, need to be tailored at a different level during undergraduate education as the expected skill sets and/or the required level of these skills may be different. I agree with other reviewers that adding references will further strengthen the manuscript.

Reviewer Expertise:

NA

No decision status is available

# Reviewer report: Reviewer response for version 1

**DOI:** 10.21956/mep.19377.r28386

**Published:** 2017-10-24

**Authors:** Paul Duggan

**Affiliations:** 1 The University of Adelaide

This review has been migrated. The reviewer awarded 2 stars out of 5 I enjoyed reading this passionate, stirring opinion piece and the earlier reviewers' comments. I believe many of us share some of the author's concerns about OSCE's. Some of those can be addressed by ensuring best quality OSCE questions (the examples mentioned highlight that) and an appropriate range of assessment items beyond OSCE's. In relation to WBA, whilst widely used in postgraduate settings, in many institutions the examiners in those settings are the same as in the medical schools. In our school, WBA's are used in the 6th (final) year and OSCE's conclude at the end of the 5th (penultimate) year. We do not have the resources to replace our summative OSCE's in 5th year with reliable high-stakes WBA's. I suspect we are not isolated in that respect, though acknowledge that in a large metropolitan area such as London those resource constraints might be less of an issue. Accepting that "assessment drives learning", the author did not mention that the medical students have a responsibility to address their own learning, in particular by seeing as many real patients as they can and identifying their own strengths and weaknesses resulting from those real clinical encounters.

Reviewer Expertise:

NA

No decision status is available

# Reviewer report: Reviewer response for version 1

**DOI:** 10.21956/mep.19377.r28384

**Published:** 2017-10-23

**Authors:** Trevor Gibbs

**Affiliations:** 1 AMEE

This review has been migrated. The reviewer awarded 2 stars out of 5 As someone too who has grown up with OSCEs and can remember the time before OSCEs, I was drawn to read and review this paper. Although I agree that we have to be very careful how we use OSCEs and at what point in time in students' life, to actually make a statement that OSCEs are outdated and encourage strategic OSCE thinking is as far as I can tell from research completely unfounded. There is nothing wrong with a correctly structured OSCE, performed and organised in a correct manner and used to specifically assess students at a specific point in their career. OSCE's do not give you a real picture of how the students perform but they do have good predictive validity Graham et. al J Dent Educ. 2013 Feb;77(2):161-7)My argument is not the value of the OSCE but the way in which it is used or misused, and specificaly how it fits into the toolbox of assessment, and how it is used with other forms of assessment to give as near as possible realistic picture of the student.I would have preferred to have seen some more evidence supporting this very strong statement

Reviewer Expertise:

NA

No decision status is available

# Reviewer report: Reviewer response for version 1

**DOI:** 10.21956/mep.19377.r28376

**Published:** 2017-10-23

**Authors:** Julie Browne

**Affiliations:** 1 Cardiff University School of Medicine

This review has been migrated. The reviewer awarded 4 stars out of 5 I thoroughly enjoyed this article and found myself almost wanting to jump to my feet and cheer at its rousing call for ‘real life’ educational experiences and assessment practices that accept the variability of genuine clinical experience rather than attempting to homogenise it. I am sure many other medical educators who have struggled for some time to encourage their students to get out of the simulation suite and onto the wards will agree with its principal premise: that if you examine students through simulation then that’s how they will prefer to learn, because “assessment drives learning”.Furthermore, I was pleased that its argument was rational and proportionate: it outlined the many unintended consequences of an over reliance on OSCEs while freely admitting the enormous challenges involved in reducing the supremacy of OSCEs as the default assessment method, especially for large scale, high stakes tests of clinical skills. I am looking forward to seeing educators rising to the challenge it poses – namely, how to develop real life assessments that can offer sufficient validity and reliability to ensure both student learning and patient safety.Just a final point - I wonder why this article has no references? It would be strengthened considerably if it were properly situated within the existing debates around simulation, workplace based assessment and education for patient safety. I would, for example, have expected to see reference to one of the earliest papers that kicked off this whole debate: Bligh J, Bleakley A. Distributing menus to hungry learners: can learning by simulation become simulation of learning? Med Teach. 2006 Nov;28(7):606-13; but there have been many more in the 12 years since it was first published.

Reviewer Expertise:

NA

No decision status is available

# Reviewer report: Reviewer response for version 1

**DOI:** 10.21956/mep.19377.r28382

**Published:** 2017-10-23

**Authors:** Megan Anakin

**Affiliations:** 1 University of Otago

This review has been migrated. The reviewer awarded 2 stars out of 5 I was interested in reading this article because I come from an education background and I, too, have experienced how powerful assessment can be as a driver of student learning. In addition to the suggestions made by the previous reviewers, a discussion of how the different principles of assessment are at play – and potentially creating the set of tensions that the author and student experienced would enhance article. The vivid anecdote about the student and persuasive argument could be augmented by referencing the literature examining students’ experiences of assessment generally, and summative medical assessments, in particular. The author may find that there is a paucity of medical education literature in this area and the need to investigate this issue further may strengthen his argument.

Reviewer Expertise:

NA

No decision status is available

# Reviewer report: Reviewer response for version 1

**DOI:** 10.21956/mep.19377.r28378

**Published:** 2017-10-23

**Authors:** Wolf Hautz

**Affiliations:** 1 Inselspital Bern

This review has been migrated. The reviewer awarded 2 stars out of 5 This is an interesting viewpoint, that elaboratly argues why OSCEs should be replaced with workplace based assessments. The authors key reason is, that - in his opinion - the OSCEs lack clinical validity and foster learning the wrong thing. Both arguments are worth consideration in my opinion.However, in advocating the replacement of OSCEs with WPBAs, the auther negelcts the very different use these assement formats have. OSCEs are most ofte used for summative purposes and are thus required to be highly reliable (which, depending on their design and the number of stations used, they often are). WPBAs are mostly - and I would add hopefully - used for formative assessment, i.e. providing feedback. As such, they should be valid, while reliability is less of a concern. Thus, OSCEs are mainly an assessment tool, while WPBAs are basically teaching tools. Replacing one with the other would mean to use the wrong tool for the task.

Reviewer Expertise:

NA

No decision status is available
